# Systematic review of risk prediction models for surgical site infection after abdominal surgery in adults

**DOI:** 10.3389/fpubh.2026.1721423

**Published:** 2026-03-10

**Authors:** Yating Xu, Juecen Liu, Yao Chen, Meixuan Song, Xianrong Li

**Affiliations:** 1 School of Nursing, Southwest Medical University, Luzhou, Sichuan, China; 2 Department of Gastrointestinal Surgery, Affiliated Hospital of Southwest Medical University, Luzhou, Sichuan, China

**Keywords:** abdominal surgery, prediction model, risk prediction model, surgical site infection, systematic review

## Abstract

**Objective:**

To systematically review risk prediction models for surgical site infection (SSI) after abdominal surgery and to provide a reference for clinical risk management.

**Methods:**

A comprehensive search was conducted in Web of Science, Cochrane Library, PubMed, Sinomed, Chinese Medical Journal Full-text Database, CNKI, VIP, and Wanfang Data for studies published from January 1, 1980, to August 12, 2024. Two researchers independently screened the literature, extracted data, and assessed the risk of bias and applicability of the models.

**Results:**

A total of 25 studies were included, involving 28 SSI risk prediction models after abdominal surgery. Among them, 25 models showed good predictive performance (AUC > 0.7), but all studies exhibited a high risk of bias. The most frequently included predictors were surgical duration, diabetes, BMI (body mass index), serum albumin levels, ASA (American Society of Anesthesiologists) physical status score, age, intraoperative blood loss, wound classification, and open surgery.

**Conclusion:**

Risk prediction models for SSI after abdominal surgery are still in the developmental stage. Future studies should emphasize model construction and validation to improve their clinical utility and generalizability.

**Systematic review registration:**

https://www.crd.york.ac.uk/PROSPERO/view/CRD42024576543, Identifier: CRD42024576543.

## 1 Introduction

Surgical site infection (SSI) is a common postoperative complication and represents an important component of hospital-acquired infections. According to the 1999 guidelines for the prevention of surgical site infection issued by the Centers for Disease Control and Prevention (CDC), SSI is defined as an infection occurring within 30 days after surgery—or within 1 year if an implant is placed—that affects the incision or deep tissues and is associated with the surgical procedure (1). As one of the most common healthcare-associated infections, SSI poses a serious threat to patient outcomes and quality of life. Abdominal surgery carries an especially high risk of SSI due to factors such as abundant subcutaneous fat, extensive operative fields, prolonged operative time, and the presence of a complex intestinal microbiota, with reported incidence rates ranging from 7.5 to 26.7% (2, 3). SSI not only exacerbates patient suffering and delays recovery but also markedly increases healthcare costs and mortality risk (4, 5). Evidence indicates that among general surgery patients, SSI is associated with an average increase of approximately 10,114.5 CNY in hospitalization expenses, a prolonged hospital stay by 13 days, and a 2- to 11-fold higher risk of death compared with uninfected patients (6, 7). Importantly, up to 60% of SSIs are preventable through evidence-based infection control measures (7). Therefore, preoperative identification of high-risk patients and implementation of tailored interventions are crucial for reducing SSI incidence.

In recent years, numerous predictive models for postoperative SSI in abdominal surgery have been developed worldwide to facilitate early risk assessment. However, considerable heterogeneity exists across these models in terms of methodological quality, predictive performance, and clinical applicability, and no systematic review has yet synthesized and compared their overall performance. To address this gap, the present study aims to systematically evaluate risk prediction models for postoperative SSI in patients undergoing abdominal surgery, with the goal of providing clinicians with robust tools for risk stratification and offering a foundation for the refinement and broader application of such models.

## 2 Materials and methods

### 2.1 Problem formulation

This study was conducted in accordance with the TRIPOD-SRMA reporting guideline for systematic reviews of prediction models (8). The research question was defined using the PICOTS framework proposed by the Cochrane Prognosis Methods Group (9):

① P (Population): adult patients (≥18 years) undergoing abdominal surgery;

② I (Intervention model): development, validation, or updating of a prediction model;

③ C (Comparator): none;

④ O (Outcome): occurrence of SSI;

⑤ T (Timing): within 30 days postoperatively;

⑥ S (Setting): inpatient settings where models are applied to predict postoperative SSI.

### 2.2 Inclusion and exclusion criteria

#### 2.2.1 Inclusion criteria

(1) Study population: adult patients (≥18 years) undergoing abdominal surgery;

(2) Content: development of a risk prediction model for postoperative SSI;

(3) Study design: cross-sectional, case–control, or cohort studies;

(4) Outcome: incidence of SSI;

(5) Language: Chinese or English.

#### 2.2.2 Exclusion criteria

(1) Duplicate publications;

(2) Studies not involving prediction model development;

(3) Models developed solely from systematic reviews or meta-analyses;

(4) Studies including fewer than two predictors;

(5) Animal studies, narrative reviews, conference abstracts, and similar publications;

(6) Studies with unavailable full texts;

(7) Dissertations or theses.

This review was registered in PROSPERO (CRD42024576543).

### 2.3 Literature search strategy

Electronic databases including Web of Science, Cochrane Library, PubMed, Sinomed, Chinese Biomedical Literature Database (CBM), CNKI, VIP, and Wanfang were systematically searched from January 1, 1980, to August 12, 2024, to identify studies on prediction models for postoperative SSI following abdominal surgery.

Chinese search terms included: abdomen, stomach, intestine, hepatobiliary, pancreas and spleen, abdominal hernia, gynecology and obstetrics, urology, surgical site infection, surgical wound infection, incision infection, risk prediction model, risk score and nomogram.

English search terms included: abdomen, Abdom, gastrointestinal, colorectal, hepatopancreatobiliary, laparotomy, gynecolog*, urolog*, gastr*, liver, surgical wound infection, surgical site infection (SSI), incision infection, prediction model, risk prediction model, risk score, nomogram, risk stratification, prognos* model, forecasting model*.

### 2.4 Study selection and data extraction

Two reviewers independently screened the literature and extracted data in accordance with the predefined eligibility criteria. Any discrepancies were resolved through consultation with a third reviewer. Data extraction was performed using a standardized form designed with reference to the checklist for systematic reviews of prediction models proposed by Moons et al. (10).

### 2.5 Quality assessment

The methodological quality of included studies was appraised using the Prediction Model Risk of Bias Assessment Tool (PROBAST) (11), which evaluates both risk of bias and applicability. Risk of bias was assessed across four domains: participants, predictors, outcomes, and analysis. Applicability was judged with respect to participants, predictors, and outcomes. Each item was rated as yes, probably yes, no, probably no, or unclear. Overall risk of bias was categorized as low, high, or unclear. A domain was considered high risk if any item was rated no or probably no. A study was deemed low risk only if all domains were judged to be at low risk. Quality assessment was independently conducted by two reviewers, and disagreements were resolved by a third reviewer.

## 3 Results

### 3.1 Literature search

**Figure 1:**
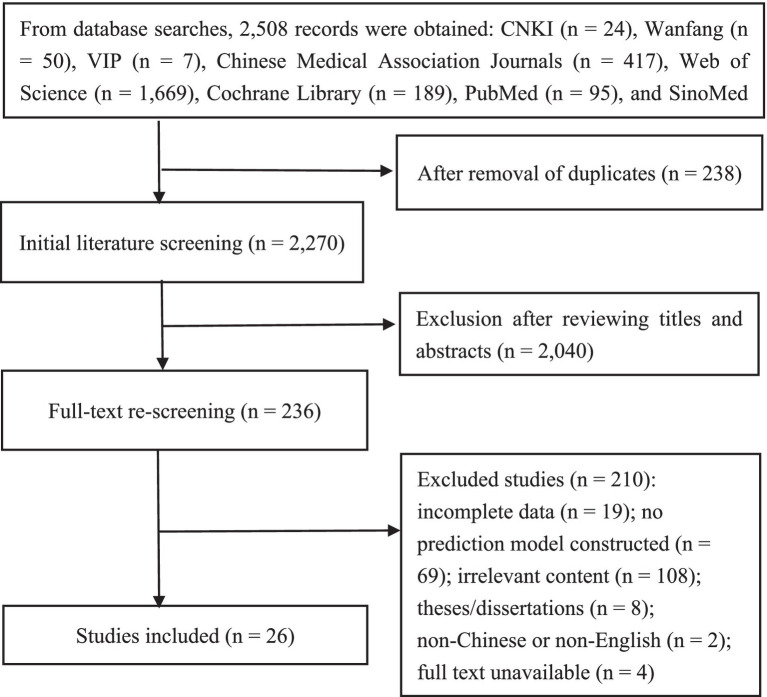
Flowchart of literature screening.

A total of 2,508 records were initially retrieved. After stepwise screening, 26 studies involving 315,858 patients undergoing abdominal surgery were included. The study selection process is presented in Figure 1.

### 3.2 Characteristics of included studies

A total of 26 studies were included, of which seven (12–18) were published in the past 3 years. Most studies were conducted in China (*n* = 20) (12–31), while the remaining six (32–37) originated from Brazil, Switzerland, the United States, Canada, and Japan. The sample size of individual studies ranged from 85 to 315,858 patients, and the number of predictors included in the models ranged from 3 to 11. Three studies (24, 32, 33) were prospective cohort studies, while the remaining 23 (12–23, 25–31, 34–37) were retrospective cohort studies. The basic characteristics of the included studies are summarized in Table 1.

**Table 1:** Basic characteristics of the included studies.

Included study	Year	Country	Model type	Study population	Study design	Data source	Total sample size			Outcome indicator	SSI observation period
							Outcome event	Total cases	Incidence rate		
de Oliveira et al. (32)	2006	Brazil	a	Patients undergoing gastrointestinal surgery	A	Clinical data	149	609	24.50%	SSI	From admission to 30 days postoperatively
Gervaz et al. (33)	2012	Switzerl	a + b	Patients undergoing colorectal resection	A	Clinical data	114	534	21.30%	SSI	From admission to 30 days postoperatively
Hedrick et al. (34)	2013	The United States	a + b	Patients undergoing elective intra-abdominal colorectal surgery	B	ACS NSQIP	1719	18,403	9.34%	Superficial/deep SSI	From admission to 30 days postoperatively
Tu et al. (19)	2016	China	a	Patients undergoing laparoscopic gastrectomy	B	Electronic medical record (EMR) system	131	2,364	5.50%	SSI	From admission to 30 days postoperatively
Li et al. (20)	2017	China	a + b	Patients undergoing hepatectomy with hepaticojejunostomy	B	Electronic medical record (EMR) system	34	335	10.15%	Space/organ SSI	Within 30 days postoperatively
Liu et al.(21)	2017	China	a + b	Patients undergoing abdominal surgery	B	Hospital information system (HIS)	362	5,067	7.14%	SSI	—
Zhao et al. (22)	2018	China	a + b	Patients undergoing abdominal surgery	B	Clinical data	84	2,931	2.87%	Surgical incision infection	—
Tianyu et al. (23)	2019	China	a + c	Patients undergoing radical hepatectomy for hepatocellular carcinoma	B	Electronic medical record (EMR) system	149	640	23.20%	SSI	—
Dang et al. (35)	2020	Canada	a + c	Patients undergoing laparoscopic Roux-en-Y gastric bypass/laparoscopic sleeve gastrectomy	B	MBSAQIP	1841	274,187	0.70%	SSI	Within 30 days postoperatively
Okui et al. (36)	2020	Japan	a + b	Patients undergoing gastrointestinal or hepatobiliary tract cancer resection	B	Electronic medical record (EMR) system	106	1,578	6.70%	Space/organ SSI	—
Kamada et al. (37)	2021	Japan	a	Patients undergoing elective stoma reversal	B	Medical records	53	182	29.00%	Superficial/deep SSI	Within 30 days postoperatively
Sun et al. (24)	2021	China	a	Patients undergoing radical resection for gastrointestinal cancer	A	RCT	51	839	6.10%	Space/organ SSI	Within 30 days postoperatively
Lin et al. (25)	2021	China	a + b	Patients undergoing surgery for gastrointestinal perforation	B	Clinical data	53	170	31.20%	Surgical incision infection	—
Wang et al. (26)	2021	China	a + c	Patients undergoing colorectal cancer surgery	B	Clinical data	18	85	21.17%	SSI	—
Pei et al. (27)	2022	China	a	Patients undergoing radical colorectal cancer surgery	B	Clinical data	46	402	11.40%	Intra-abdominal infection	Within 30 days postoperatively
Bu et al.(28)	2022	China	a + b	Patients undergoing colorectal cancer surgery	B	Clinical data	40	413	9.70%	SSI	Within 30 days postoperatively
Gao et al. (29)	2022	China	a + b	Patients undergoing emergency surgery for intestinal obstruction	B	Clinical data	38	204	18.63%	Surgical incision infection	—
Huang et al. (30)	2022	China	a + b	Patients undergoing radical gastrectomy	B	Clinical data	66	355	18.60%	SSI	Within 30 days postoperatively
Xu et al. (31)	2022	China	a + b	Patients undergoing elective surgery for gastrointestinal/hepatobiliary/pancreatic tumors	B	Clinical data	94	982	9.60%	Space/organ SSI	—
Zhang et al. (12)	2023	China	a	Patients undergoing abdominal surgery	B	Hospital information system (HIS)	150	3,018	5%	SSI	—
Fu et al. (13)	2023	China	a	Patients undergoing radical colorectal cancer surgery	B	Electronic medical record (EMR) system	45	242	18.60%	Surgical incision infection	—
Li et al. (14)	2023	China	a	Patients undergoing colorectal cancer surgery	B	Electronic medical record (EMR) system	34	398	8.54%	Surgical incision infection	—
Miao et al. (15)	2023	China	a + c	Patients with colon cancer complicated by intestinal obstruction	B	Clinical data	143	833	17.16%	Surgical incision infection	Within 30 days postoperatively
Wang et al. (16)	2023	China	a	Patients undergoing colorectal cancer surgery	B	Clinical data	26	104	25%	Surgical incision infection	—
Xu et al. (17)	2023	China	a + b	Patients undergoing surgery for gastric and colorectal cancer	B	Electronic medical record (EMR) system	gastric cancer 30; colorectal cancer 29	gastric cancer 356; colorectal cancer 305	gastric cancer 8.4%; colorectal cancer 9.5%	Space/organ SSI	Within 30 days postoperatively
Yu et al. (18)	2024	China	a + b	Patients undergoing radical gastrectomy	B	Clinical data	27	322	8.30%	Intra-abdominal infection	—

a, Development of the prediction model; b, internal validation of the model; c, external validation of the model; A, prospective cohort study; B, retrospective cohort study; —, not reported.

### 3.3 Characteristics of risk prediction models

In total, 31 prediction models for postoperative SSI following abdominal surgery were identified. The reported AUC values ranged from 0.64 to 0.933, with 28 models achieving an AUC greater than 0.70, indicating good discriminatory ability. Ten studies (13–15, 20, 21, 26, 29, 31, 35, 36) reported both specificity and sensitivity, with specificity ranging from 52.17 to 99.95% and sensitivity ranging from 48 to 93.55%. Fifteen models (15, 16, 18, 19, 21, 24–32, 34) assessed calibration, while four studies (15, 23, 26, 35) performed external validation. Internal validation was reported in 14 studies (17–22, 25, 28–31, 33, 34, 36), most commonly using bootstrapping or split-sample methods (See Table 2 for details). The number of predictors included per model ranged from 3 to 11. Frequently reported predictors were operative time, diabetes, body mass index (BMI), serum albumin level, American Society of Anesthesiologists (ASA) score, age, intraoperative blood loss, incision type, and open surgery (see Table 3).

**Table 2:** Predictive model performance and presentation formats.

Included study	Model performance			Model validation method	Model presentation
	AUC (model development/internal validation/external validation)	Calibration method	Specificity/sensitivity/accuracy		
de Oliveira et al. (32)	0.732/—/—	Hosmer–Lemeshow test	—	—	Risk index/risk score scale
Gervaz et al. (33)	0.729/0.703/—	—	—	Internal cross-validation	Risk scoring system
Hedrick et al. (34)	Model 1.(0.64/—/—) Model 2.(0.69/—/—) Model 3.(0.65/—/—)	Calibration plot	—	Internal validation (bootstrap)	Nomogram
Tu et al. (19)	0.734/—/—	Hosmer–Lemeshow test	—	Internal random split validation	Risk scoring system
Li et al. (20)	0.851/0.850/—	—	74.1%/85.3%/—	Internal validation (bootstrap)	Nomogram
Liu et al.(21)	0.803/—/—	Hosmer–Lemeshow test	67.99%/74.83%/68.49%	Internal random split validation	—
Zhao et al. (22)	0.735/—/—	—	—	Internal validation (bootstrap)	Nomogram
Tianyu et al. (23)	0.85/0.86/0.84	—	—	External validation	Nomogram
Dang et al. (35)	0.74/—/0.73	—	99.95%/48%/—	External validation	Risk scoring formula with *β* coefficients
Okui et al. (36)	POD1all:0.751/—/— POD3all:0.883/—/— POD5all:0.818/—/—	—	POD3all:81%/85%/—	Internal cross-validation	Nomogram
Kamada et al. (37)	0.773/—/—	—	—	—	Risk scoring system
Sun et al. (24)	0.78/—/—	Hosmer–Lemeshow test	—	—	Risk stratification
Lin et al. (25)	0.834/—/—	Calibration curve	—	Internal validation (bootstrap)	Nomogram
Wang et al. (26)	0.869/—/—	Hosmer–Lemeshow test	74%/76%/—	External validation	Risk scoring formula with *β* coefficients
Pei et al. (27)	0.931/—/—	Calibration curve	—	—	Nomogram
Bu et al.(28)	0.862/0.873/—	Calibration curve	—	Internal random split validation	Nomogram
Gao et al. (29)	0.786/—/—	Hosmer–Lemeshow test	66.87%/84.21%/—	Internal validation	Risk scoring formula with *β* coefficients
Huang et al. (30)	0.826/—/—	Calibration curve	—	Internal validation (bootstrap)	Nomogram
Xu et al. (31)	0.754/0.708/—	Hosmer–Lemeshow test	52.17%/93.55%/—	Internal random split validation	Nomogram
Zhang et al. (12)	0.926/—/—	—	—	—	Nomogram
Fu et al. (13)	0.806/—/—	—	73.6%/80%/—	—	Tree diagram
Li et al. (14)	0.905/—/—	—	85.62%/87.72%/—	—	Risk scoring formula with *β* coefficients
Miao et al. (15)	0.892/—/0.786	Hosmer–Lemeshow test	80.77%/92.31%/—	External validation	Nomogram
Wang et al. (16)	0.928/—/—	Hosmer–Lemeshow test	—	—	Risk scoring formula with *β* coefficients
Xu et al. (17)	Model 1.(0.808/—/—) Model 2.(0.763/—/—)	—	—	Internal validation (bootstrap)	Nomogram
Yu et al. (18)	0.933/0.951/—	Hosmer–Lemeshow test	—	Internal validation (bootstrap)	Nomogram

—: Not reported.

**Table 3:** Model development status.

Included studies	Modeling method	Number of models	Missing data		Method for handling continuous variables	Number of predictors	Final included predictors
			Number	Handling method			
de Oliveira et al. (32)	Logistic regression	1	36	—	Categorical variables	4	Obesity, surgical risk, adjusted operative time, laparoscopic surgery
Gervaz et al. (33)	Logistic regression	1	—	—	Categorical variables	4	Obesity, wound classification III-IV, open surgery, ASA score III-IV
Hedrick et al. (34)	Logistic regression	3	—	Multiple imputation	Continuous variables	8	Open surgery, BMI, preoperative hematocrit, ASA score, smoking, alcohol use, preoperative functional status, age > 75 years
Tu et al. (19)	Logistic regression	1	—	—	Categorical variables	4	BMI, intraoperative blood loss, operative time, perioperative transfusion
Li et al. (20)	Logistic regression	1	—	—	Categorical variables	4	Bile duct stones, intraoperative blood loss >1,500 mL, history of abdominal surgery, bile leakage
Liu et al.(21)	Logistic regression	1	—	—	Categorical variables	8	Laparoscopic surgery, wound classification, operative time, diabetes, hypoalbuminemia, hypertension, preoperative inflammatory response, ASA score
Zhao et al. (22)	Logistic regression	1	—	Deletion	Categorical variables	5	Diabetes, age, malnutrition, emergency surgery, lack of postoperative health education
Tianyu et al. (23)	Logistic regression	1	—	—	Continuous variables	4	ASA score, operative time, serum albumin level, repeat hepatectomy
Dang et al. (35)	Logistic regression	1	—	—	Categorical variables	9	BMI, operative time, diabetes, hypertension, Roux-en-Y gastric bypass, long-term use of corticosteroids or immunosuppressants, sex, gastroesophageal reflux disease, obstructive sleep apnea
Okui et al. (36)	Logistic regression	3	47/54/108	Deletion	Continuous variables	5	White blood cell count, platelet count, albumin, C-reactive protein, estimated glomerular filtration rate
Kamada et al. (37)	Logistic regression	1	—	—	Continuous variables	3	Subcutaneous fat thickness ≥20 mm, stoma duration ≤20 weeks, SSI after primary surgery
Sun et al. (24)	Logistic regression	1	—	—	Categorical variables	5	Gastrectomy, colorectal resection, pancreatoduodenectomy, anesthesia time >4 h, prolonged ICU stay
Lin et al. (25)	Logistic regression	1	—	—	Categorical variables	5	Operative time ≥2 h, unwashed incision, BMI ≥ 24 kg/m^2^, no preoperative antibiotics, intraoperative incision extension
Wang et al. (26)	Logistic regression	1	—	—	Categorical variables	9	BMI, operative time, diabetes, wound classification, age, serum albumin level, Dukes stage, type of surgery, probiotic supplementation
Pei et al. (27)	Logistic regression	1	—	—	Categorical variables	4	Preoperative serum albumin level, lymphocyte-to-white blood cell ratio < 0.17, low subcutaneous fat mass, low skeletal muscle mass
Bu et al.(28)	Logistic regression	1	—	—	Categorical variables	6	Open surgery, diabetes, male sex, BMI ≥ 28 kg/m^2^, preoperative moderate anemia, preoperative neoadjuvant chemotherapy
Gao et al. (29)	Logistic regression	1	—	—	Categorical variables	3	Surgery duration ≥ 2 h, age ≥ 65 years, albumin < 28 g/L
Huang et al. (30)	Logistic regression	1	—	—	Categorical variables	8	BMI, surgery duration, diabetes, age, body temperature upon entering the operating room, intermittent warming time, intraoperative lowest temperature, surgical approach
Xu et al. (31)	Logistic regression	1	—	—	Categorical variables	5	White blood cell count, neutrophil-to-lymphocyte ratio, total bilirubin, C-reactive protein, mean body temperature
Zhang et al. (12)	Logistic regression	1	—	—	Categorical variables	11	Antibiotic use, NRS 2002 score ≥ 3, NNIS score ≥ 2, procalcitonin ≥ 0.05 μg/L, low-density lipoprotein < 3.37 mmol/L, general anesthesia, blood loss ≥ 200 mL, surgical site, wound class III, season of surgery, diabetes
Fu et al. (13)	Decision tree model	1	—	—	Categorical variables	5	Intraoperative blood loss, surgery duration, diabetes, serum albumin level, TNM stage
Li et al. (14)	Logistic regression	1	—	—	Categorical variables	3	Hypoproteinemia, BMI ≥ 24 kg/m^2^, surgery duration ≥ 3 h
Miao et al. (15)	Logistic regression	1	—	—	Categorical variables	6	Open surgery, diabetes, surgery duration ≥ 2 h, age ≥ 60 years, postoperative indwelling catheter ≥ 3 days, neoadjuvant chemotherapy
Wang et al. (16)	Logistic regression	1	—	—	Continuous variables	2	Diabetes, drainage tube indwelling time
Xu et al. (17)	Logistic regression	2	—	—	Categorical variables	4/3	Gastric cancer: ASA score, intraoperative blood loss, sex, combined organ resection; Colorectal cancer: ASA score, surgery duration, combined organ resection
Yu et al. (18)	Logistic regression	1	—	—	Continuous variables	6	BMI, intraoperative blood loss, surgery duration, serum albumin level, blood glucose level, hemoglobin

Note: —: Not report.

### 3.4 Quality assessment of included studies

#### 3.4.1 Risk of bias assessment

All studies were judged to be at high risk of bias, indicating methodological shortcomings in both the model development and validation phases (see Table 4).

**Table 4:** Inclusion of literature quality assessment.

Included studies	Risk of bias				Applicability			Overall	
	Participants	Predictors	Outcome	Data analysis	Participants	Predictors	Outcome	Risk of bias	Applicability
de Oliveira et al. (32)	−	?	+	+	+	+	+	+	+
Gervaz et al. (33)	+	−	−	+	+	+	+	+	+
Hedrick et al. (34)	+	?	+	+	+	+	+	+	+
Tu et al. (19)	+	?	?	+	+	+	+	+	+
Li et al. (20)	+	?	?	+	+	+	+	+	+
Liu et al.(21)	+	+	+	+	+	+	+	+	+
Zhao et al. (22)	+	?	?	+	+	+	+	+	+
Tianyu et al. (23)	+	+	+	+	+	+	+	+	+
Dang et al. (35)	−	+	?	+	+	+	+	+	+
Okui et al. (36)	+	+	+	+	+	+	+	+	+
Kamada et al. (37)	+	+	+	+	+	+	+	+	+
Sun et al. (24)	−	?	?	+	+	+	+	+	+
Lin et al. (25)	+	+	+	+	+	+	+	+	+
Wang et al. (26)	+	+	+	+	+	+	+	+	+
Pei et al. (27)	+	+	?	+	+	+	+	+	+
Bu et al.(28)	+	+	?	+	+	+	+	+	+
Gao et al. (29)	+	+	+	+	+	+	+	+	+
Huang et al. (30)	+	+	?	+	+	+	+	+	+
Xu et al. (31)	+	?	?	+	+	+	+	+	+
Zhang et al. (12)	+	+	+	+	+	+	+	+	+
Fu et al. (13)	+	+	+	+	+	+	+	+	+
Li et al. (14)	+	+	+	+	+	+	+	+	+
Miao et al. (15)	+	+	?	+	+	+	+	+	+
Wang et al. (16)	+	+	?	+	+	+	+	+	+
Xu et al. (17)	+	+	?	+	+	+	+	+	+
Yu et al. (18)	+	+	?	+	+	+	+	+	+

—, indicates low risk of bias or low applicability; +, indicates high risk of bias or high applicability;?, indicates unclear.

In the participants domain, 23 studies (12–23, 25–31, 34–37) were retrospective in design and therefore considered at high risk of bias. Five studies (12, 21, 33, 34, 36) applied relatively broad inclusion and exclusion criteria, leading to high risk of bias.

In the predictors domain, 22 studies (12–21, 23, 25–30, 32, 34–37) lacked sufficient information to determine whether predictor definitions and assessment criteria were consistent across participants, resulting in unclear risk of bias. In addition, 21 studies (12–18, 21–31, 35–37) did not clarify whether predictor assessors were blinded, also leading to unclear risk.

In the outcomes domain, four studies (23, 32, 34, 37) applied inappropriate SSI classifications and were judged to be at high risk of bias. Another four (13, 14, 21, 26) did not report classification methods, and three (12–14) failed to define SSI explicitly, all rated as unclear risk. One study (23) applied an unreasonable SSI definition, rated as high risk. Furthermore, 23 studies (12–31, 35–37) did not specify whether outcome assessors were blinded, resulting in unclear risk of bias.

In the analysis domain, nine studies (16, 18, 20, 25, 26, 29, 32, 33, 37) had inadequate sample sizes for model development, rated as high risk. Two studies (32, 33) inappropriately handled continuous and categorical predictors, and 18 studies (12–18, 21–23, 25–29, 34, 35, 37) lacked sufficient information regarding variable handling, leading to high risk. Three studies (31, 32, 36) directly excluded cases with missing data, considered high risk, while 20 studies (12–16, 18–29, 33, 35, 37) did not report missing data at all, indicating inadequate reporting. In addition, 21 studies (12–15, 17–20, 22–25, 27–30, 32, 33, 35–37) relied on univariate analyses for predictor selection, regarded as high risk. Eleven studies (12–14, 17, 20, 22, 23, 33, 35–37) did not report model calibration methods, resulting in unclear risk. Six studies (13, 19, 20, 22, 34, 36) did not assess model updating or adjustment, rated as high risk. Fourteen studies (12, 14–16, 18, 23, 24, 26, 27, 29, 31, 32, 35, 37) did not report model fit issues, leaving the risk unclear.

#### 3.4.2 Applicability assessment

All studies focused on adult patients (≥18 years) undergoing abdominal surgery, and the target populations were consistent across studies, indicating good applicability.

## 4 Discussion

Surgical site infection (SSI) is one of the most common complications following abdominal surgery and is closely associated with patient mortality, complication risk, and healthcare costs (38). Developing accurate risk prediction models can facilitate early identification of high-risk patients and enable timely interventions, thereby reducing the incidence of SSI. Among the 31 models included in this study, most demonstrated satisfactory predictive performance (AUC > 0.7); however, significant deficiencies remain in model development and reporting quality, highlighting the need for further optimization.

### 4.1 Risk prediction models for post-abdominal surgery SSI are still in an exploratory stage

Currently, the development of SSI risk prediction models after abdominal surgery remains preliminary, with several methodological limitations. First, among the 26 included studies, most were single-center, and only three (24, 32, 33) were prospective; the remainder were retrospective, which may limit the reliability and validity of the datasets. Second, many studies did not rigorously adhere to blinding procedures during predictor assessment and outcome evaluation, potentially introducing bias and affecting model accuracy. Additionally, according to sample size principles for predictive modeling, each predictor variable should typically include 10–20 outcome events (39); however, nine studies (16, 18, 20, 25, 26, 29, 32, 33, 37) had insufficient sample sizes.

Furthermore, variable selection methods remain largely traditional, often relying on univariate analysis, which may omit important predictors or include irrelevant variables, compromising model robustness. Novel approaches such as LASSO regression, SelectKBest, and genetic algorithms (GA) have demonstrated higher efficiency in various modeling contexts (40) and could be considered in future studies. Most studies also failed to report methods for handling missing values and predictor variables; three studies (31, 32, 36) excluded missing data directly, and 18 studies (12–18, 21–23, 25–29, 34, 35, 37) provided no information regarding predictor handling, resulting in a high risk of bias that may impair model generalizability.

Finally, only four studies (15, 23, 26, 35) conducted external validation. The lack of external validation in most models makes it difficult to assess whether their predictive performance is stable, thereby limiting generalizability and clinical applicability. Moreover, the external validation cohorts in most studies were drawn from the same institutions as the development cohorts, emphasizing reproducibility but not model transportability (41). Although some studies reported discrimination (e.g., AUC or C-index), calibration (e.g., Hosmer–Lemeshow test), and clinical utility, overall performance reporting was insufficient, and some models may be overfitted, restricting broader application. Most predictive models included in this study primarily reported the area under the receiver operating characteristic curve (AUC), sensitivity, and specificity as their main performance metrics, whereas indicators better suited for imbalanced outcomes, such as the area under the precision–recall curve (AUC-PR) and F1 score, were rarely reported. Only a few studies further evaluated the clinical utility or overall predictive accuracy of the models. For instance, Bu et al. (28) employed decision curve analysis (DCA) to assess the net clinical benefit of the model, while Huang et al. (30) reported a Brier score of 1.14 to reflect the overall agreement between predicted probabilities and observed outcomes. In summary, risk prediction models for post-abdominal surgery SSI are still in an early developmental stage, and future efforts should comprehensively improve data sources, modeling design, validation strategies, and reporting quality.

### 4.2 Heterogeneity in SSI incidence due to variations in definitions and diagnostic criteria

The incidence of surgical site infection (SSI) reported in the included studies ranged from 0.7 to 31.2%, indicating substantial heterogeneity across studies, likely attributable to differences in SSI definitions, classification, and diagnostic criteria. A standardized international definition of SSI is currently lacking, and most studies have referenced the prevention guidelines issued by the Centers for Disease Control and Prevention (CDC) (1). Among the included studies, 20 were conducted in China, where SSI was primarily defined according to the 2001 Chinese Ministry of Health’s “Diagnostic Criteria for Hospital Infection” (42). While the core principles of this guideline are largely consistent with the CDC’s 1999 “Guidelines for the Prevention of Surgical Site Infection,” discrepancies remain in specific classifications and implementation details.

Of the 26 included studies, 10 studies (17, 19, 20, 23, 24, 32–34, 36, 37) adopted the CDC SSI diagnostic criteria; 4 studies (12, 27, 28, 35) mentioned SSI definitions without specifying the reference standard; 6 studies (14–16, 21, 22, 29) used the 2001 Chinese Ministry of Health criteria; 4 studies (13, 25, 26, 31) did not report SSI definitions; 1 study (30) referred to the 2018 Asia Pacific Infection Control guidelines, and 1 study (18) followed the Chinese Society of Gastrointestinal Cancer guidelines. These variations in SSI definitions and diagnostic standards may compromise the comparability and consistency of reported outcomes. Moreover, SSI can be further classified into superficial incisional, deep incisional, organ/space, and intra-abdominal infections, each with inherently different risk profiles and incidence patterns.

The incidence of SSIs may vary according to the surgical site, surgical approach, and surgical technique. Surgical technique (open vs. minimally invasive) is an independent determinant of infection risk; however, most existing predictive models have not adequately accounted for this variable, neither including it as a key predictor nor validating model performance in subgroups defined by surgical approach. It is also important to note that the timing of SSI assessment differs by procedure; for example, in patients with implants, the monitoring period may extend up to one year postoperatively. However, most studies included in this analysis did not explicitly report whether such procedures were included or adjust the assessment period accordingly, which may have led to misclassification of some infection events. In addition, most studies relied on retrospective data collection from hospital databases or electronic medical records. Differences in healthcare resources, economic conditions, cultural factors, and infection surveillance systems across countries and regions may further influence the accuracy of SSI monitoring and diagnosis. Some studies also lacked detailed descriptions of SSI definitions, diagnostic procedures, or surveillance methods, limiting the reproducibility of results and cross-study comparability.

Based on the factors described above, future studies should further standardize SSI outcome assessment, particularly by clearly specifying whether implant-related procedures are included and defining the corresponding monitoring period. Well-designed prospective studies are warranted to improve the accuracy and consistency of outcome measurement. On this basis, SSI risk prediction models should be further validated and refined for different populations and subgroups, including those with and without implants.

### 4.3 Predictors of SSI following abdominal surgery

This study summarized and analyzed the commonly used predictors in existing models, identifying operative duration, diabetes, body mass index (BMI), serum albumin level, ASA score, age, intraoperative blood loss, incision type, and open surgery as consistently high-frequency risk factors for postoperative SSI following abdominal surgery. These variables are generally easy to measure and readily accessible in clinical practice, enhancing their practical applicability.

Zeitlinger et al. (42) reported a positive correlation between operative time and SSI risk, with longer surgeries associated with higher infection rates, potentially due to factors such as surgical complexity, prolonged tissue exposure, and immunosuppression. Bhat et al. (43) suggested that surgeries exceeding 90 min constitute an independent risk factor for SSI, whereas Shen et al. (44) indicated that operative durations ≥180 min are associated with SSI. These findings highlight ongoing debate regarding specific time thresholds, warranting further investigation. Therefore, optimizing preoperative preparation, carefully planning surgical procedures, minimizing operating room personnel movement, and reducing operative time can mitigate risks of instrument contamination and tissue necrosis, ultimately decreasing SSI incidence. Additionally, organizing training sessions and competitions to enhance surgical knowledge and technical proficiency among staff can shorten operative duration and reduce patient harm.

Diabetes, as a chronic metabolic disorder, significantly increases postoperative infection risk due to immune dysfunction and microcirculatory impairment (45). Clinical practice should emphasize perioperative blood glucose monitoring for diabetic patients, with timely intervention for abnormal glucose levels to maintain relative glycemic stability. Particular attention should be paid to early postoperative fasting periods and parenteral nutrition, such as lipid emulsions, to manage glucose levels. Systematic patient education should also be implemented, addressing the psychological state of patients, informing them and their families about diabetes-related risks and dietary considerations, and encouraging active participation in self-management of blood glucose.

Elevated BMI is recognized as an independent risk factor for SSI, as excessive subcutaneous fat can compromise wound perfusion and contribute to tissue necrosis, thereby increasing infection risk (46). Bone et al. (47) found that a BMI > 25 kg/m^2^ increased SSI risk in patients undergoing hepatobiliary surgery, while another study reported BMI > 24 kg/m^2^ as an independent risk factor for SSI following cesarean section (48). BMI thresholds may vary according to surgical type. For elective surgery candidates, preoperative lifestyle interventions such as moderate exercise and dietary adjustments are recommended. Postoperatively, close monitoring of wound status, encouragement of early ambulation, and maintenance of stable glucose levels and body weight are essential.

Serum albumin is an important indicator of nutritional status, and malnutrition reduces resistance and immune function. Hypoalbuminemia can impair wound healing and adversely affect postoperative outcomes (49). Nutritional assessment upon admission, using laboratory measures such as serum albumin and validated assessment scales, is essential. When malnutrition is identified, communication with patients is necessary, alongside dietary adjustments and, when required, enteral or parenteral nutritional support.

ASA score reflects the patient’s preoperative comorbidities and overall physical condition, with higher scores typically associated with chronic conditions such as diabetes and cardiovascular disease, which may compromise immunity and increase infection risk (50). For patients with ASA class III or higher, surgical teams should actively coordinate with anesthesiologists to optimize patient status, ensure thorough preoperative preparation, adhere strictly to aseptic protocols, and reinforce postoperative care to reduce SSI risk.

Increasing age is associated with declining immune function and a higher prevalence of comorbidities, which correspondingly raises infection risk (51). For older patients with multiple comorbidities and poorly controlled conditions, detailed preoperative counseling and interventions to enhance immune function and manage underlying diseases are essential to minimize SSI occurrence. Wang et al. (52) reported that each 1 mL increase in intraoperative blood loss elevated SSI risk by 0.2%, potentially due to increased transfusion-related susceptibility and intraoperative contamination. Surgical teams should employ meticulous techniques to minimize tissue trauma, maintain effective hemostasis, and carefully consider transfusion indications to reduce SSI risk.

Studies have found that Class III incisions carry higher infection rates than Class II, highlighting the importance of proactively managing preoperative infection for higher-risk procedures (53). Surgical approach also significantly affects SSI risk; laparoscopic surgery, associated with smaller incisions and faster recovery, substantially reduces SSI risk compared to open procedures (54). Liu et al. (55) reported SSI rates of 20.86% for open surgery versus 8.61% for laparoscopic surgery. Preoperative patient assessment should guide selection of the most appropriate surgical approach, with laparoscopic procedures preferred when feasible to reduce trauma, postoperative infection risk, and promote recovery.

Gender was also identified as a factor influencing SSI, consistent with findings by Xu et al. (17). Most studies suggest that males have higher SSI risk due to greater abdominal fat, denser body hair, and higher rates of smoking and alcohol consumption, with male colorectal cancer patients exhibiting a 1.2-fold higher SSI risk than females (50, 56). Conversely, Pedroso-Fernandez et al. (57) indicated that females may prefer smaller incisions for cosmetic reasons, potentially increasing surgical difficulty and SSI risk. Some studies, however, report no significant association between gender and postoperative SSI (58). The relationship between gender and SSI remains controversial and warrants further large-scale, high-quality investigations.

### 4.4 Implications for future predictive model development

To enhance the scientific rigor and clinical utility of SSI risk prediction models following abdominal surgery, future studies should consider optimization in the following areas: (1) Diversifying predictor sources: incorporating readily available clinical and laboratory indicators; (2) Standardizing blinding and reporting: improving model transparency and reproducibility; (3) Rigorous model development design: adopting an EPV ≥ 20 criterion and a minimum sample size of 100 for validation; (4) Standardized variable handling: clearly defining cut-off values for continuous variables and reporting methods for handling missing data; (5) Optimizing variable selection methods: incorporating machine learning techniques such as LASSO regression to enhance model performance; (6) Strengthening validation strategies: employing multicenter study designs, expanding population diversity, and improving model generalizability and transportability; (7) Visual and practical implementation: translating models into web-based calculators, scoring systems, or integrating them into electronic medical record systems for convenient clinical use.

Additionally, adherence to reporting standards such as TRIPOD is recommended, encompassing model development, internal and external validation, and clinical evaluation, to systematically report the research process. This approach can enhance transparency and quality of reporting and promote the advancement of risk prediction model research.

### 4.5 Limitations

This study has several limitations. First, the reported SSI incidence varied considerably among the included studies, likely due to differences in definitions, classification, and diagnostic criteria, and the varying risk profiles of different SSI types may also affect comparability. Most studies were retrospective and conducted in different countries or regions with diverse infection surveillance and diagnostic practices, which may further compromise outcome ascertainment. Second, only studies published in English and Chinese were included, which could introduce publication bias and potentially omit high-quality studies in other languages. Furthermore, most predictive models were developed and validated in China, limiting their generalizability. Heterogeneity in sample size and predictor variables across studies precluded meta-analysis, restricting the precision of quantitative synthesis. In addition, most models primarily reported AUC, sensitivity, and specificity, while performance metrics more suitable for imbalanced outcomes, such as AUC-PR and F1 score, were seldom reported, and internal and external validations were generally lacking, indicating room for improvement in model development and evaluation. Future research should aim to standardize SSI definitions, adopt prospective study designs, and incorporate multidimensional performance metrics along with established model development and reporting guidelines, such as PROBAST and TRIPOD, to enhance model reliability and clinical applicability.

## 5 Conclusion

This study reviewed 28 predictive models for postoperative SSI in adult abdominal surgery. The systematic evaluation revealed that existing models still have notable limitations, including inconsistent SSI definitions and diagnostic criteria, high heterogeneity of data sources, and insufficient methodological quality and reporting standards. These shortcomings reduce the accuracy and generalizability of the models. Future research should focus on standardized SSI outcome assessment, rigorous methodological design, transparent reporting, and multicenter validation to enhance the clinical utility of predictive models, advance SSI risk prediction, and ultimately improve patient quality of life.

## Data Availability

The original contributions presented in the study are included in the article/supplementary material, further inquiries can be directed to the corresponding author.

## References

[1] Mangram AJ Horan TC Pearson ML Silver LC Jarvis WR. Guideline for prevention of surgical site infection, 1999. Centers for Disease Control and Prevention (CDC) hospital infection control practices advisory committee. Am J Infect Control. (1999) 27:97–132. 10196487

[2] Alkaaki A Al-Radi OO Khoja A Alnawawi A Alnawawi A Maghrabi A . Surgical site infection following abdominal surgery: a prospective cohort study. Can J Surg. (2019) 62:111–7. doi: 10.1503/cjs.004818, 30907567 PMC6440888

[3] Marzoug OA Anees A Malik EM. Assessment of risk factors associated with surgical site infection following abdominal surgery: a systematic review. BMJ Surg Interv Health Technol. (2023) 5:e000182. doi: 10.1136/bmjsit-2023-000182, 37529828 PMC10387634

[4] K B R S J S Satyanesan J. Surgical site infections in gastrointestinal surgeries: estimation of prevalence, risk factors and bacteriological profile. Cureus. (2024) 16:e62589. doi: 10.7759/cureus.62589, 39027770 PMC11256213

[5] Savio L Simeone P Baron S Antonini F Bruder N Boussen S . Surgical site infection in severe trauma patients in intensive care: epidemiology and risk factors. Ann Intensive Care. (2024) 14:136. doi: 10.1186/s13613-024-01370-7, 39218984 PMC11366732

[6] Wang X Lan T Chang X Yang X. Distribution of pathogenic bacteria and analysis of infection risk factors in surgical site infections of colorectal cancer patients. J Pathog Biol. (2023) 18:1214–1217+1222. doi: 10.13350/j.cjpb.231020

[7] Liu S Zhang N Sun J Liu Y Yang L Xu X. Interpretation of the implementation plan of special action "strengthen perioperative infection prevention and control and ensure surgical quality and safety". Chin J Nosocomiol. (2024) 34:3521–5. doi: 10.11816/cn.ni.2024-241185

[8] Snell KIE Levis B Damen JAA Dhiman P Debray TPA Hooft L . Transparent reporting of multivariable prediction models for individual prognosis or diagnosis: checklist for systematic reviews and meta-analyses (TRIPOD-SRMA). BMJ. (2023) 381:e073538. doi: 10.1136/bmj-2022-073538, 37137496 PMC10155050

[9] Wang Z Lu C Zhang J Huang J Liu W Shang W . Interpretation of checklist for transparent reporting of multivariable prediction models for individual prognosis or diagnosis tailored for systematic reviews and meta-analyses (TRIPOD-SRMA). Chin J Evid Based Med. (2024) 24:202–10.

[10] Moons KG de Groot JA Bouwmeester W Vergouwe Y Mallett S Altman DG . Critical appraisal and data extraction for systematic reviews of prediction modelling studies: the CHARMS checklist. PLoS Med. (2014) 11:e1001744. doi: 10.1371/journal.pmed.1001744, 25314315 PMC4196729

[11] Moons KGM Wolff RF Riley RD Whiting PF Westwood M Collins GS . Probast: a tool to assess risk of bias and applicability of prediction model studies: explanation and elaboration. Ann Intern Med. (2019) 170:W1–w33. doi: 10.7326/m18-1377, 30596876

[12] Zhang J Xue F Liu SD Liu D Wu YH Zhao D . Risk factors and prediction model for inpatient surgical site infection after elective abdominal surgery. World J Gastrointest Surg. (2023) 15:387–97. doi: 10.4240/wjgs.v15.i3.387, 37032800 PMC10080607

[13] Fu G Zhao Y Zheng Y Wu K Li L Wang L. Etiological characteristics of postoperative incision infections in patients undergoing radical resection of colorectal cancer and establishment of predictive decision tree model. Chin J Nosocomiol. (2023) 33:3270–4. doi: 10.11816/cn.ni.2023-230400

[14] Li W Zheng X Sun B Zhang H Zhu Z Zhao E. Risk factors for postoperative incision infection in colorectal cancer patients and establishment of prediction model. Chin J Nosocomiol. (2023) 33:2652–5. doi: 10.11816/cn.ni.2023-230109

[15] Miao F Zhu X Liu Z Zhang H Li N Zhang C. Pathogen distribution, influencing factors and nomogram prediction model of postoperative incision infection in patients with colon cancer and intestinal obstruction. J Pathog Biol. (2023) 18:336–41. doi: 10.13350/j.cjpb.230318

[16] Wang C Huang H Si C Yang D Ma Y. Risk factors for postoperative incision infection in colorectal cancer and logistic regression model. Chin J Nosocomiol. (2023) 33:3104–7. doi: 10.11816/cn.ni.2023-230357

[17] Xu Y Fei W Gao L Yang J. Analysis of risk factors and construction of prediction models for organ/space surgical site infection in gastric and colorectal cancer. Chin J Infect Control. (2023) 22:1003–12. doi: 10.12138/j.issn.1671-9638.20234162

[18] Yu X Tang W Bai C Li R Feng B Wu J . A predictive model for intraabdominal infection after radical gastrectomy in elderly patients. Medicine. (2024) 103:e37489. doi: 10.1097/md.0000000000037489, 38489739 PMC10939676

[19] Tu RH Huang CM Lin JX Chen QY Zheng CH Li P . A scoring system to predict the risk of organ/space surgical site infections after laparoscopic gastrectomy for gastric cancer based on a large-scale retrospective study. Surg Endosc. (2016) 30:3026–34. doi: 10.1007/s00464-015-4594-y, 26487214 PMC4912586

[20] Li L Ding J Han J Wu H. A nomogram prediction of postoperative surgical site infections in patients with perihilar cholangiocarcinoma. Medicine. (2017) 96:e7198. doi: 10.1097/md.0000000000007198, 28640107 PMC5484215

[21] Liu X He W Huang X Sun J Li W Wu A . Risk prediction model for abdominal surgical site infections. Chin J Nosocomiol. (2017) 27:132–5. doi: 10.11816/cn.ni.2016-161978

[22] Zhao L Wang F Hu J. Nomogram model of predicting postoperative surgical wound infection in patients with abdominal surgery. Chin J Mod Nurs. (2018) 14:1633–8. doi: 10.3760/cma.j.issn.1674-2907.2018.14.007

[23] Tang TY Zong Y Shen YN Guo CX Zhang XZ Zou XW . Predicting surgical site infections using a novel nomogram in patients with hepatocelluar carcinoma undergoing hepatectomy. World J Clin Cases. (2019) 7:2176–88. doi: 10.12998/wjcc.v7.i16.2176, 31531313 PMC6718804

[24] Sun C Gao H Zhang Y Pei L Huang Y. Risk stratification for organ/space surgical site infection in advanced digestive system cancer. Front Oncol. (2021) 11:705335. doi: 10.3389/fonc.2021.705335, 34858805 PMC8630667

[25] Lin X Guo S Zhou D Guan Y. Establishment of a nomogram model for predicting the risk of postoperative wound infection in patients with gastrointestinal perforation. Chin J Infect Chemother. (2021) 21:669–74. doi: 10.16718/j.1009-7708.2021.06.006

[26] Wang Y Chen C Wu T Lin M Wang R. Risk factors for surgical site infection in colorectal cancer patients and establishment of prediction model. Chin J Nosocomiol. (2021) 31:663–7. doi: 10.11816/cn.ni.2021-202420

[27] Pei G Zhen S Zhang B. A novel nomogram based on nutritional and immune status predicting postoperative intra-abdominal infection in colorectal cancer. Asia Pac J Clin Nutr. (2022) 31:626–35. doi: 10.6133/apjcn.202212_31(4).0006, 36576281

[28] Bu N Zhao S Wang B Gao Y Gao W. A nomogram based on preoperative hemoglobin levels for the prediction of postoperative surgical site infection in patients with colorectal cancer. Int J Anesthesiol Resusc. (2022) 2:151–8. doi: 10.3760/cma.j.cn321761-20210723-00478

[29] Gao F You D Li J Zhang S Qian Q Wang W. Risk factors for incision infection after intestinal obstruction surgery and construction of prediction model. Chin J Nosocomiol. (2022) 32:3605–8. doi: 10.11816/cn.ni.2022-212380

[30] Huang D He L Li L. Construction of a prediction model for postoperative surgical site infection of gastric cancer. Chin Nurs Manag. (2022) 22:1519–24. doi: 10.3969/j.issn.1672-1756.2022.10.017

[31] Xu J Jiang Y Yu E Yang X. Construction and validation of risk prediction model for surgical site infection in organ/lacuna of postoperative patients with cancer of gastrointestinal, liver, pancreatic, bile duct. Chin Nurs Res. (2022) 36:2497–502. doi: 10.12102/j.issn.1009-6493.2022.14.010

[32] de Oliveira AC Ciosak SI Ferraz EM Grinbaum RS. Surgical site infection in patients submitted to digestive surgery: risk prediction and the NNIS risk index. Am J Infect Control. (2006) 34:201–7. doi: 10.1016/j.ajic.2005.12.011, 16679177

[33] Gervaz P Bandiera-Clerc C Buchs NC Eisenring MC Troillet N Perneger T . Scoring system to predict the risk of surgical-site infection after colorectal resection. Br J Surg. (2012) 99:589–95. doi: 10.1002/bjs.8656, 22231649

[34] Hedrick TL Sawyer RG Friel CM Stukenborg GJ. A method for estimating the risk of surgical site infection in patients with abdominal colorectal procedures. Dis Colon Rectum. (2013) 56:627–37. doi: 10.1097/DCR.0b013e318279a93e, 23575403

[35] Dang JT Tran C Switzer N Delisle M Laffin M Madsen K . Predicting surgical site infections following laparoscopic bariatric surgery: development of the BariWound tool using the MBSAQIP database. Surg Endosc. (2020) 34:1802–11. doi: 10.1007/s00464-019-06932-6, 31236724

[36] Okui J Ueno R Matsui H Uegami W Hayashi H Miyajima T . Early prediction model of organ/space surgical site infection after elective gastrointestinal or hepatopancreatobiliary cancer surgery. J Infect Chemother. (2020) 26:916–22. doi: 10.1016/j.jiac.2020.04.009, 32360091

[37] Kamada T Ito E Ohdaira H Takahashi J Takeuchi H Kitagawa K . New scoring system for predicting the risk of surgical site infections following stoma reversal. J Surg Res. (2021) 267:350–7. doi: 10.1016/j.jss.2021.05.041, 34198111

[38] Bahru TT Gobena MA Anteneh BT Meskelu HA Legese AT Berhanu HT . Prevalence of surgical site infection and associated factors among post-operative patients. Int Wound J. (2025) 22:e70730. doi: 10.1111/iwj.70730, 40685844 PMC12277540

[39] Chen Y Yu W Gao J Chen X Cheng Q Cui J. Predictive value of TyG-BMI, AIP, and postprandial glucose excursion for sarcopenia in patients with type 2 diabetes mellitus. J Army Med Univ. (2025) 47:1792–9. doi: 10.16016/j.2097-0927.202505021

[40] Yang B Bai Y Lang L Cao Q Zhu G Zang L . Risk factors for postoperative respiratory failure in patients with esophageal cancer and the prediction model establishment. Chin J Clin Thorac Cardiovasc Surg. (2025) 32:353–9. doi: 10.7507/1007-4848.202407020

[41] Zhou X Sun J Wang X Deng H Wang L Zhu L . Interpretation of and insights from the guidelines for external validation of clinical risk prediction models. J Nurs Sci. (2024) 39:52–6. doi: 10.3870/j.issn.1001-4152.2024.24.052

[42] Zeitlinger L Wilson M Randall RL Thorpe S. Surgical duration is independently associated with an increased risk of surgical site infection and may not be mitigated by prolonged antibiotics: a secondary analysis of the PARITY trial of infection after lower-extremity Endoprosthetic reconstruction for Bone tumors. J Bone Joint Surg Am. (2023) 105:79–86. doi: 10.2106/jbjs.23.00056, 37466584

[43] Bhat RA Isaac NV Joy J Chandran D Jacob KJ Lobo S. The effect of American Society of Anesthesiologists score and operative time on surgical site infection rates in major abdominal surgeries. Cureus. (2024) 16:e55138. doi: 10.7759/cureus.55138, 38558689 PMC10979762

[44] Shen Y Hu YL Xu JH Zhu S Cai L Wu YF . Incidence, risk factors, outcomes, and prediction model of surgical site infection after hepatectomy for hepatocellular carcinoma: a multicenter cohort study. Eur J Surg Oncol. (2025) 51:109486. doi: 10.1016/j.ejso.2024.109486, 39615293

[45] Martin ET Kaye KS Knott C Nguyen H Santarossa M Evans R . Diabetes and risk of surgical site infection: a systematic review and meta-analysis. Infect Control Hosp Epidemiol. (2016) 37:88–99. doi: 10.1017/ice.2015.249, 26503187 PMC4914132

[46] Farhan SA Farhan SH Janis JE. Does BMI impact outcomes in patients undergoing open abdominal wall reconstruction? A systematic review and meta-analysis. World J Surg. (2025) 49:1777–1786. doi: 10.1002/wjs.12649, 40528287 PMC12282558

[47] Bone M Latimer S Walker RM Thalib L Gillespie BM. Risk factors for surgical site infections following hepatobiliary surgery: an umbrella review and meta-analyses. Eur J Surg Oncol. (2025) 51:109468. doi: 10.1016/j.ejso.2024.109468, 39579465

[48] Li L Cui H. The risk factors and care measures of surgical site infection after cesarean section in China: a retrospective analysis. BMC Surg. (2021) 21:248. doi: 10.1186/s12893-021-01154-x, 34011324 PMC8132410

[49] Xie J Liu H Deng S Niu T Wang J Wang H . Association between immediate postoperative hypoalbuminemia and surgical site infection after posterior lumbar fusion surgery. Eur Spine J. (2023) 32:2012–9. doi: 10.1007/s00586-023-07682-9, 37027034

[50] Xu Z Qu H Kanani G Guo Z Ren Y Chen X. Update on risk factors of surgical site infection in colorectal cancer: a systematic review and meta-analysis. Int J Color Dis. (2020) 35:2147–56. doi: 10.1007/s00384-020-03706-8, 32748113

[51] Ke C Dong X Xiang G Zhu J. Risk factors and nomogram predictive model of surgical site infection in closed pilon fractures. J Orthop Surg Res. (2023) 18:582. doi: 10.1186/s13018-023-04058-z, 37553679 PMC10408134

[52] Wang A Yuan S Zhou Y Su X Kong W Chen L . Analysis of risk factors for spinal surgical site infections and establishment and validation of a column-line diagram. J Jinan Univ (Nat Sci Med Ed). (2024) 45:51–9. doi: 10.11778/j.jdxb.20230206

[53] Gonzalez KW Dalton BG Kurtz B Keirsey MC Oyetunji TA St Peter SD. Operative wound classification: an inaccurate measure of pediatric surgical morbidity. J Pediatr Surg. (2016) 51:1900–3. doi: 10.1016/j.jpedsurg.2016.07.010, 27530888

[54] Koike T Mukai M Kishima K Yokoyama D Uda S Hasegawa S . The association between surgical site infection and postoperative colorectal cancer recurrence and the effect of laparoscopic surgery on prognosis. Langenbeck's Arch Surg. (2024) 409:40. doi: 10.1007/s00423-024-03234-x, 38225456

[55] Liu X Peng L Zeng L. Role of ASA score and operation time in risk assessment of surgical site infection in patients with colorectal cancer. Chinese Journal of Infection Control. (2021) 20:1144–1148. doi: 10.12138/j.issn.1671-9638.20211423, 37553679

[56] Li Z Wen S Wang Z Wu C Wang P Ren J . Multicenter cross-sectional study of surgical site infection after emergency surgery in China. Chin J Pract Surg. (2019) 39:1052–6. doi: 10.19538/j.cjps.issn1005-2208.2019.10.14

[57] Pedroso-Fernandez Y Aguirre-Jaime A Ramos MJ Hernández M Cuervo M Bravo A . Prediction of surgical site infection after colorectal surgery. Am J Infect Control. (2016) 44:450–4. doi: 10.1016/j.ajic.2015.10.024, 27038393

[58] Zwicky SN Gloor S Tschan F Candinas D Demartines N Weber M . No impact of sex on surgical site infections in abdominal surgery: a multi-center study. Langenbeck's Arch Surg. (2022) 407:3763–9. doi: 10.1007/s00423-022-02691-6, 36214869 PMC9722878

